# Assessment of Exposure to Sexually Explicit Materials and Substance Abuse among High-School Adolescents in North Shewa Zone: Application of Logistic Regression Analysis

**DOI:** 10.1155/2020/8105087

**Published:** 2020-05-09

**Authors:** Berhanu Teshome Woldeamanuel, Leul Mekonnen Anteneh, Yordanos Berihun Yohannes, Merga Abdissa Aga

**Affiliations:** Department of Statistics, College of Natural and Computational Sciences, Salale University, Fitche, Ethiopia

## Abstract

**Background:**

The use of substances such as cigarettes, khat, alcohol, and other illicit drugs like hashish, heroine, cannabis, and cocaine is a global major public threat, which affects young adult people particularly in developing countries. This study aims to assess the risk factors associated with substance use and exposure to sexually explicit materials among high-school adolescents in north Shewa zone, Oromia region.

**Method:**

A cross-sectional study was conducted to assess substance use and exposure to sexually explicit materials among high-school adolescents in North Shewa zone, Oromiya, Ethiopia, using a structured self-administered questionnaire adapted from the 2008 “Community That Care Youth Survey” for adolescent substance use and problem behaviors. The study used descriptive statistics and logistic regression analysis to identify the significant factors associated with substance use and exposure to sexually explicit materials among high school adolescents in the study area.

**Result:**

The prevalence of lifetime and current substance use was 47.7% (95% CI: 0.427, 0.527) and 30.4% (95% CI: 0.258, 0.350), respectively. 17.8% use khat in their life and 16.6% used khat in the past 30 days; 42.2% ever used alcohol and 26.1% currently uses alcohol; 4.8% and 4.5% used cigarette in lifetime and in the past 30 days, while 16.4% use other illicit drugs in lifetime and 8.4% use illicit drugs in the past 30 days, respectively. Distributions of substance use by sex indicate that male adolescents are more like likely 61.1% use substances than females. While, the prevalence of exposure to sexually explicit materials among high school adolescents was 35.8% (95% CI: 0.310, 0.406). Factors positively associated with increased substance use were being male (OR = 2.334, 95% CI: 1.549, 9.926), living through high level of family conflict (OR = 6.25, 95% CI: 1.745, 10.00), poor family management OR = 27.084, 95% CI: 1.624, 45.56), peer pressure (OR = 12.882, 95% CI: 1.882, 88.153), poor academic performance (OR = 14.48, 95% CI: 1.290, 162.58), and low school commitment (OR = 11.951, 95% CI: 1.418, 100.73). While, being male (OR = 7.52, 95% CI: 2.611, 21.739), age 14–16 (OR = 0.201, 95% CI: 0.071, 0.565), friends watch/read sexually explicit materials (OR = 5.376, 95% CI: 1.010, 28.571), and khat chewing (OR = 12.5, 95% CI: 2.924, 25.632) were factors significantly associated with high-school adolescents exposure to sexually explicit materials.

**Conclusion:**

The magnitude of prevalence for substance uses and exposure to sexually explicit materials in the study area was still higher. Therefore, interventions that focus on family management, peer pressure, and school commitment are required to decrease the prevalence of substance uses and exposure to sexually explicit materials among high-school adolescents.

## 1. Introduction

The use of substances such as cigarettes, khat, alcohol, and other illicit drugs like hashish, heroine, cannabis, and cocaine is a global major public threat, which affects young adult people particularly in developing countries [1–3]. Drug uses disorders and ill-health-related conditions are major public health problems. Worldwide, it is estimated that alcohol and illicit drugs accounted for 5.4% and 3.7% of the global burden of disease in 2009, respectively [4]. It is also estimated that 9% of the world population aged 12 or older are classified with dependence on psychoactive substances such as alcohol [5].

The United Nations in 2014 indicated globally, alcohol consumption accounts for about 5.9% or nearly 3.3 million deaths of the global deaths, and 5.1% of the global burden of disease and injury were attributable to alcohol consumption in 2012 [6]. Early start of drinking increases the likelihood of alcohol-related injuries, motor vehicles crashes involvement, unprotected intercourse, and interpersonal violence. Alcohol uses also to contribute to youth suicides, homicides, and fatal injuries [7].

The most locally produced and highly consumed alcohols in Ethiopia are tella, areque (katikala), shamita, tej, and borde. Aside from locally fermented alcohols beers, gin, wine, and other alcoholic liquors are widely used alcohols products [8]. The Ethiopian public health association (EPHA) in 2007 reported that about 30.5% of adolescents aged 15–24 in Ethiopia ever used substances in their life, while young adults seem to be the most involved in alcohol and khat consumption than any other population group [9]. A similar study based on the 2005 behavioral surveillance surveys have reported that males are more likely to use substances than females, but the trends for the habits of using substances is rising from time to time among females [10]. The Ethiopian Demographic and Health Survey 2016 reported the prevalence of alcohol use among men and women is 46% and 35%, respectively, and 27% of men and 12% of women ever chewed khat [11].

Previous studies showed that peer pressure, accessibility, cultural accessibility, parental involvement, and low self esteem are the major reasons for starting using substances in Ethiopia, while some adolescents use substances to relax, and to avoid or forget their problems and anxieties, like problems arising from economic stress [12].

A cross-sectional study conducted on substance uses and associated factors among Haramaya university students indicated that more than 62% of students use at least one substance, 50.2% use alcohol, 41% chewed khat in their lifetime, 23.6% currently use khat, while 22% were using cigarettes at least once in their lifetime, 10.8% currently smoke cigarette and 7.4% used illicit drug like hashish [13]. A systematic review and meta-analysis on substance use among adolescents aged 10–19 years in sub-Saharan Africa between 2000 and 2016 revealed the prevalence of substance use ranged from 37% in South Africa to 55.5% in central Africa with the overall prevalence of substance use 41.6%. About 32.8% uses alcohol, 23.5% uses cigarette, 22% use khat, and 15.9% use cannabis [14].

Wakgari and Aklilu studied substance use and its predictors among undergraduate medical students of Addis Ababa University in Ethiopia, the study reported 22% of students used alcohol, 7% used khat, and 9% of students use cigarette [15]. A similar study conducted in Debre Birhan University reported 36.3% of the lifetime use of alcohol, 10.9% lifetime use of khat, and 7.4% lifetime use of cigarette among students, while 4.2% and 4.5% of students were used shisha and cannabis, respectively, in their lifetime. Further, the study reported 17%, 5.7%, and 3.1% of students are currently using alcohol, khat, and cigarette, respectively [16]. Shimelis and Wosen in their study on causes, prevalence, and consequences of alcohol and drug abuse among Mekelle University students reported the prevalence of alcohol and drug abuse among students is 30% and the major reason for alcohol and drug abuse are peer pressure, psychological factors, academic factors, and social factors [17].

A study conducted in Nepal reported, 6% prevalence of substance use among high-school adolescents [18]. A similar study in the Southeast Asian nations reported that the lifetime prevalence of illicit drug uses was 16.9% [19].

Another study conducted in Gondar University showed 62.9% prevalence of drug uses and the major contributing factors of substance uses are relations, increase performance, and peer pressure [20]. While Gobeje et al. [21] in their studies about the prevalence of substance use and associated factors among preparatory students of Woldia Town, northeast Ethiopia, in 2015 found that the overall prevalence of substance use is 34.6%, whereas 23.5% of students chewed khat and use cigarettes. A multidomain factor analysis studies conducted in Woreta Town found 47.9% prevalence of current substance uses and 65.4% prevalence of lifetime substance use among high-school adolescents in Woreta Town [22]. Similarly, a study done on prevalence of substance abuses among University students in Tigray, northern Ethiopia, reported 43.9% of university students abuse substances [23].

In Harar, eastern Ethiopia, Reda [24] found a 12.2% prevalence of cigarette smoking among school adolescents. While, the Drug Administration and Control Authority in 2005 found 10.1%–11.5% prevalence of a lifetime smoking and 3%–5.6% current prevalence of cigarette smoking among high-school adolescents in Addis Ababa [25].

*Exposure to sexually explicit materials (SEM)* means a picture, photograph, drawing, sculpture, motion picture film, or similar visual representation which is obscene for children. Pornography and SEM will be used interchangeably. Webster's dictionary defines pornography as “the depiction of erotic behavior intended to cause sexual excitement.” Exposure to sexually explicit materials stands viewing sexually explicit material through media such as the Internet, videos, and magazines that may be directly linked with the sexual behavior of adolescents and young adults. Globally, adolescents spend more time with the media than they do in school or with their parents [26]. Much of what young people are listening to and/or watching includes sexual content. Males have been found to be more likely to expose themselves to SEMs than females. In Ethiopia, youths aged 15–24 years were more than 15.2 million, contributing to 20.6% of the whole population [27]. These large and productive groups of the population are exposed to various sexual and reproductive health risks. Among many sexual and reproductive health risks, sexual coercion, early marriage, polygamy, unplanned pregnancies, closely spaced pregnancies, abortion, and sexually transmitted infections (STIs) are the major ones [28].

Risk and protective factors for youth substance abuse should be assessed as a prevention program as they help decrease unhealthy behavior [29, 30]. Individual protective factors, such as engagement in positive meaningful activities, positive self-concept, and religious or spiritual beliefs (religiosity) inhibit adolescent substance use. Peer protective factors, such as positive peer role models [31] also reduce adolescent substance use. Family factors that are found to be protective for adolescent substance use are connected to family (attachment/bonding), positive parenting style, living in a two parent family, higher parent education, and higher parental expectations about school. School protective factors include being connected to school (attachment/bonding), and caring school climate, and community protective factors are connected to other positive adults (bonded/attached), safe, supportive, connected neighborhoods, and community rewards for prosocial involvement [32].

Uses of substance and its consequence is one of the recent problems aggravate adolescents. The sustainable development goal (SDG) indicator, 3.5 targeted to strengthen the prevention and treatment of substance abuse, including narcotic drug abuse and harmful use of alcohol by the year 2030 [33].

**Figure 1:**
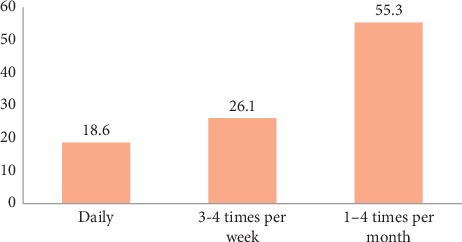
Frequency of watching sexually explicit materials.

**Figure 2:**
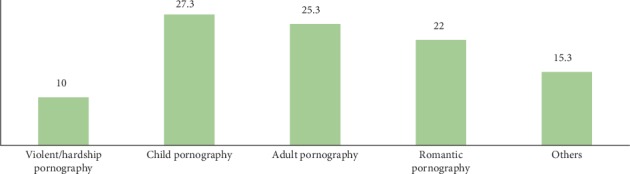
Percentage of sexually explicit materials consumed by most adolescents.

Despite the fact that a number of researches have been done on substance uses, nowadays in Ethiopia many adolescent students and young adults migrate from rural to urban areas to attend secondary and higher educations or in search of job. This will in turn relate to risky sexual behavior. The prevalence of substance uses and exposure to risky sexual behavior is still high, and more effort is needed to minimize the rate of substance use and exposure to sexually risky materials. The finding of this study will help for the development of preventive policies, effective intervention priorities, and any cost benefit analysis-related substance abuse and exposure to SEM. It will contribute to the literature by highlighting the influential characteristics of substance abuse and exposure to SEM by various social, economic, cultural, and demographic factors. It could also be part of baseline information for policy makers. Therefore, the main objective of this study is to assess the prevalence and associated factors of substance use and exposure to sexually explicit materials among high-school adolescents in North Shewa zone of Oromia regional state, Ethiopia (Figures 1 and 2).

## 2. Methods

### 2.1. Study Area

The study was conducted from March–May, 2019 in North Shewa zone of Oromia regional state, which is located around 114 km northwest of the capital Addis Ababa, with an elevation between 2515 and 2547 metres above sea level. Based on the 2007 census conducted by the Central Statistical Agency of Ethiopia (CSA), this zone has a total population of 1,431,305, of whom 717,552 are men and 713,753 women; with a population density of 138.66. Only 10.25% are urban inhabitants. The largest ethnic group of CSA reported on this area was Oromo (84.33%) and followed by Amhara (14.99%); all other ethnic groups made up 0.68% of the population.

### 2.2. Study Design

A cross-sectional study was conducted to assess substance abuse and exposure to sexually explicit materials (SEM) among high-school adolescents in North Shewa zone, Oromiya, Ethiopia. A structured self-administered questionnaire will be adapted from the 2008 “Community That Care (CTC) Youth Survey” for adolescent substance uses and problem behaviors. Relevant modifications were made to incorporate information specific to this study and its local context. The data will be collected by trained data collectors and supervisors.

### 2.3. Target Population

The target population comprised all high-school adolescences aged 14–19 years. There were more than 10,000 high-school students in the north Shewa zone, Oromia region.

### 2.4. Study Population

All high-school adolescences were randomly selected from the target population.

### 2.5. Data Sample Size Determination

The sample size was determined using the formula of sample size determination for single population proportion considering 50% prevalence of substance use from prior similar study [21], assuming of 95% confidence interval, 5% marginal error, and 10% nonresponse rates.

(1) $$\begin{array}{c} n=\frac{{\left(z_{\alpha /2}\right)}^{2}\;pq}{d^{2}}\;, \end{array}$$

where *n* = sample size, *z*_*α*/2_ = critical value to obtained from standard normal distribution, *α* = level of significance, *p* = proportion of street children, and *d* = margin of errors:

(2) $$\begin{array}{c} n_{0}=\frac{{\left(z_{\alpha /2}\right)}^{2}\;pq}{d^{2}}\;=\frac{{\left(1.96\right)}^{2}\;0.5*0.5}{{\left(0.05\right)}^{2}}=385. \end{array}$$

Then considering 10% nonresponse rate, the total sample size was =(*n*_0_+0.1*∗n*_0_)=424 .

## 3. Sampling Procedure

### 3.1. Description of Data Collection Procedures

The survey used a two-stage simple random sampling technique. The first stage involved selecting schools in the study areas and the second stage, number of students per school was selected with a probability proportional to size. There were 14 districts in the North Shewa zone. The sampling frame was obtained from North Shewa zone education bureau. In the first stage, eight districts and one high school was selected within each district using simple random sampling technique. Accordingly the selected districts (schools) are Abichu (Mendida secondary school), Dera (Gundo meskel secondary school), Kuyu (Gebregurracha secondary school), Fiche (Abdissa Aga secondary school), Kimbibit (Sheno secondary school), Wechale (Mukaturi secondary school), Wara jarso (Tullu milki secondary school), and Debre Libanos (Derbretsige secondary school). In the second stage, a fixed number of students were selected using systematic random sampling technique. Sample size has been allocated for every grade based on proportional allocation to their size. Lastly, students from every class had been selected by systematic random sampling (*k* = 12).

### 3.2. Data Collection Procedure

The research instruments that were employed under this study adapted from the standard the 2008 “Community That Care Youth Survey” for adolescent substance use and problem behaviors and similar prior literatures. The data collectors were bachelor degree holders who have guided the students to fill the questionnaire. The data collectors briefed each question to the respondents to help them understand the question well and complete the questionnaire. The researchers have followed and facilitated the overall data collection process and trained the data collectors. The questionnaire is designed to gather qualitative and quantitative data pertaining to demographic, socioeconomic, and cultural factors and awareness and knowledge level on substance use.

### 3.3. Study Variables

The dependent variables are substance use and exposure to sexually explicit materials.

The independent variables were socio demographic characteristics (age, sex, grade, religion, mother's educational level, father's educational level, and family economic level), religiosity, friends' use of drugs/substances, poor family management (poor family discipline and poor family supervision, family conflict), family history of alcohol and substance use, poor academic performance/academic failure, low commitment to school, and perceived availability of substances.

### 3.4. Statistical Data Analysis

Descriptive statistics such as frequency distribution and cross tabulation and bar charts were used to summarize the distribution of selected characteristics of high school adolescents, while logistic regression analysis was employed to identify the statically significant factors of substance uses and exposure to sexually explicit materials among high-school adolescents.

### 3.5. Ethical Clearance

The study was undertaken after the approval of the research proposal by research and community service directorate of Salale University before the data collection started. A formal letter was written from Salale University to all the concerned authorities. For confidentiality, the name of the participants and ID number were not typed on the questionnaire. All responses were anonymous and kept confidential.

#### 3.5.1. Inclusion Criteria

- All regular high-school students in the North Shewa zone, Oromia region
- Adolescents ages between 14 and 19 years

#### 3.5.2. Exclusion Criteria

- All students less than 14 years old and adolescents greater than 19 years old

### 3.6. Operational Definition

 
*Adolescence*. The period from age 14–19 years.
 
*Substance*. The three commonly used psychoactive drugs alcohol, cigarette, and khat that produce changes in mood, thinking, feeling, and/or behavior that can cause dependence.
 
*Substance Use*. Taking any of the three commonly used psychoactive substances: alcohol, cigarette, and/or khat in the past 30 days.
 
*Risk Factors*. Characteristics or conditions within the individual or in the family, school, or community that increase the likelihood that someone will engage in the use of alcohol, cigarette, and khat or discourage positive behavior that might prevent them [5].
 
*Lifetime/Ever-Use*. Adolescents' use of the particular substance at least once in their lifetime.
 
*Current Use/30-Day Uses*. Adolescents' use of the substance at least once in the 30 days prior to data collection and is a more sensitive indicator of the level of current use of the substance.

## 4. Results

### 4.1. Description of Sample Characteristics

The total sample 424 identified 386 high school students fill the questionnaire with a response rate of 91%. About 61% of the students included were male. Slightly more than half 56.5% of the respondents were grade 10 students. The vast majorities (87.4%) of respondents were Coptic orthodox Christian and 39.2% were attending religious programs daily. About two-thirds (66%) were reported they had academic rank of 1–10 in the recent semester. 71.1% were living with their biological parents and 16.9% were living oneself. 53.7% and 62.1% of respondents were from father and mother with no formal education respectively. The age distribution of respondents showed that majority of the respondents 71.1% were in the age group 17–19 years old. The economic level of respondents' parents also varied, 64.3% of the respondents were from moderate income families, 20.5% were from poor families, and 15.2% were from families with high income status.

### 4.2. Prevalence of Substance Uses

Overall, 47.7% (95% CI: 0.427, 0.527) of the respondents reported ever used any substance and 30.4% (95% CI: 0.258, 0.350) uses any substances in the past 30 days. 17.8% (95% CI: 0.140, 0.216) use khat in their life and 16.6% (95% CI: 0.129, 0.203) uses khat in the past 30 days, 42.2% (95% CI: 0.373, 0.471) ever used alcohol and 26.1% (95% CI: 0.217, 0.305) currently uses alcohol, 4.8% (95% CI: 0.027, 0.069) and 4.5% (95% CI: 0.024, 0.066) uses cigarette in lifetime and in the past 30 days, while 16.4% (95% CI: 0.127, 0.201) use other illicit drugs in lifetime and 8.4% (95% CI: 0.056, 0.0.112) use illicit drugs in the past 30 days, respectively. Distributions of substance use by sex indicate that male adolescents more likely (61.1%) use substances than females.

Students are also asked to respond for how long they used substances. Accordingly, 35.5% used substance for shorter than six months, 26.8% uses substance for six months to one year, 21.4% of respondents used substances for one to three years and 16.1% used substances for at least three years. Regarding the question where was the first place you consumed the drugs, 71% respond at home, 8.3% reported at khat or shisha store, 4.1% in school ground, 2.4% at party, and 14.3% were started using substance at bar or restaurant and others. Additionally, respondents were asked to rate the problem of drug uses in their school; 31.8% reported the problem is very high/very serious, 9.3% said it is somewhat serious, while 7.7% reported substance use is not too serious problem in school.

### 4.3. Motivation to Use Substance

The students were also asked to respond what motivates them to use substances. Accordingly, about 9.1% use substance due to peer pressure, 13.4% due to academic failure, 15.1% to cope up with various life challenges, 18.8% to experience pleasure, and 22.5% of respondents motivated to use substance to be sociable. 30.8% of students reported they use khat to spend the leisure time, 16.9% being addicted, and 13.8% uses khat to be sociable. Similarly, 27.2% of adolescents use alcohol to pass the time, 6.4% use alcohol to leave depression, 10.4% to forget their problems, 13.6% report they use alcohol to be sociable, and 8% of the adolescents reported that they use alcohol being they are addicted.

### 4.4. Consequences of Substance Use

This study has found that substance use has various consequences. Concerning the behavioral consequences 12.5%, 15%, 16.7%, and 6.7% of respondents were experienced lateness from school, violence (disciplinary problems), absenteeism from school, and loss of interest in daily activities as a result of using substances, respectively. On other hand, regarding the social aspects, 22.6% were experienced losing friends, 39.3% experienced problems with parents/guardians and 7.1% being arrested due to substance use. Similarly, for heath related problems, 35.8% reported physically ill-health, 11.1% reported sleep disorder, and 16% experienced mental illness. From a total substance users 54.1% reported psychological distress and 11.2% were reported suicide attainment as psychological consequences of substance use. Generally, 26.9%, 21.8%, 10.3%, and 9% of respondents experienced health-related problems, social problems, psychological problems, and behavioral problems consequences of substance use in their lifetime (Tables 1–3).

### 4.5. Assessment of Goodness of Fit of the Models

We start first by checking the overall goodness of fit using the likelihood ratio test and Hosmer–Lemeshow goodness of fit test. We then proceed to test the significance of each predictor variable included in the model using the Wald test. Accordingly, the results are summarized in Table 4, and the LRT based on chi-square distribution provided a significant test value (*p* value ≤0.001) for both models. Additionally, the Hosmer–Lemeshow test revealed that there was a significance difference between the model with no covariate and the model with explanatory variables. A model with a covariate is a good fit to the data onto both models.

### 4.6. Factors Associated with Substance Use

In the multivariable logistic regression analysis, the variables sex, high level of family conflict, poor family management, age, low academic performance, substance use by friends, low level of school commitment, and mothers' education level showed a statistically significant association with adolescents' substance use.

Male adolescents were 2.334 times (OR = 2.334, 95% CI: 1.549, 9.926) more likely to use any substances than females. The odds of substance use were 27.084 times (OR = 27.084, 95% CI: 1.624, 45.56) higher among students with poor family management. Similarly, living through high levels of family conflict was positively associated with substance use (OR = 6.25, 95% CI: 1.745, 10.00) among adolescents keeping other variables constant. Adolescents whose friends use substance were more likely to use of substance than those who have friends who did not use substance (OR = 11.928, 95% CI: 1.761, 18.692). As adolescents' age is, concerned adolescents aged 14–16 were at a lower risk of substance use compared to 17–19 years old adolescents (OR = 0.261, 95% CI: 0.100, 0.683). Those adolescents who had poor academic performance were more likely to use substance (OR = 14.48, 95% CI: 1.290, 162.58) than those who performed well. The odds of substance uses were higher among adolescents who have reported low school commitment (OR = 11.951, 95% CI: 1.418, 100.73). Further peer pressure from friends was another variable that significantly associated with substance use. The odds of substance use were 12.882 times more likely (OR = 12.882, 95% CI: 1.882, 88.153) for those with peer pressure from friends than that of no peer pressure.

### 4.7. Prevalence of Exposure to SEM Uses

From the total adolescents included in the study, about 35.8% (95% CI: 0.310, 0.406) were exposed to sexually explicit materials. The major sources of sexually explicit materials were mobile phone (38.3%), video discs (14%), Internet access/search (14.5%), video houses (7%), games (5.4%), and magazines (2.8%). The mean age of adolescents at the start of watching sexually explicit materials was 15.08 with ±3.307 standard deviation (SD). Regarding frequency of watching sexually explicit materials 18.6% reported they were watching SEM daily, 26.1% watch three to four times per week while 55.3% of respondents watch such materials one to four times a month.

Concerning to the question on sexually explicit materials consumed by most adolescents, 27.3% reported child pornography, 25.3% adult pornography, and 22% romantic pornography. 24.8% of respondents were watching/read pornographic materials alone, while 43.3% and 17.7% were watching such materials with friends and families, respectively.

Regarding access to sexually explicit materials, the vast majority 61.5% of adolescents download sexually explicit materials from their mobile phone while 17.3% get from Internet cafes. As practice of SEM is concerned, 19.3% of adolescents had practiced what they had seen/read from sexually explicit materials. Concerning exposure to sexually explicit materials by sex, 71.1% adolescents exposed to sexually explicit materials were male adolescents. Among adolescents who drink alcohol, 51.4% were exposed to SEM while 33.3% and 31.9% of adolescents chewing khat and using other illicit drugs, were reported they were exposed to SEM, respectively.

### 4.8. Factors Associated with Exposure to Sexually Explicit Materials

The multivariate logistic regression analysis presented in Table 5 indicates that the odds of being exposed to SEM were 7.52 times (OR = 7.52, 95% CI: 2.611, 21.739) more likely among male students than females. Adolescents who are aged between 14 and 16 years old had a significant lower odds of being exposed to sexually explicit materials (OR = 0.201, 95% CI: 0.071, 0.565) than adolescents 17–19 years old. Adolescents whose friends watch/read SEM were 0.186 times (OR = 0.186, 95% CI: 0.035, 0.990) more likely exposed to sexually explicit materials than those whose friends did not watch SEM (Tables 6 and 7).

As family educational status is concerned, those adolescents whose father had secondary education level were 7.996 times (OR = 7.996, 95% CI: 1.010, 63.306) less exposed than those whose father had attained a diploma or higher education. Adolescents whose mother did not work and self-employed had less exposed to SEM (OR = 25.151, 95% CI: 3.290, 192.293) and (OR = 6.577, 95% CI: 1.013, 42.721), respectively. Similarly, regressing family economic status, the odds of exposure to sexually explicit materials among adolescents that belongs to a household with poor (OR = 8.433, 95% CI: 1.647, 43.18) and moderate (OR = 3.948, 95% CI: 1.088, 14.327) income levels was less than that of rich income.

## 5. Discussion

The lifetime prevalence of substance uses was 47.75% and the prevalence of current substance use is 30.4%. The finding of this study is lower than studies conducted in Haramaya university [13] that reported 62.4% of lifetime prevalence of substance use, in Woreta Town [22] that reported 65.4% prevalence of lifetime substance use and 47.9% prevalence of current substance use among high school students, a study conducted in Woldia Town [21] reported 50% prevalence of current substance, and in Kenya [34] that reported 69.8% of lifetime substance use, but higher than finding of a systematic review and meta-analysis study in sub-Saharan Africa [14] that reported 41.6% of lifetime prevalence of substance use, in Sri Lanka [35] that reported 2.7% prevalence of lifetime substance use, in India [36] that reported 12.5% prevalence of life time substance use, in Southeast Asia [19] that reported 16.9% of lifetime drug use, and in Nepal [18] that reported 6% prevalence current substance use. A study conducted in Rift Valley University [37] also reported a slightly lower 44.8% prevalence of lifetime substance use and a higher prevalence of current substance use 39.1%. The reason for this difference may be the difference in culture, accessibility of drugs, and variation in ages of study sample. In line with prior studies [13, 15, 21], this study found that the prevalence of current and lifetime substance use is higher among males.

In the current study, the prevalence of a lifetime and current alcohol use was 42.16% and 26.1%, which is congruent with a study in Uganda [38] that reported 26.8% of current alcohol users and is lower than a study done in Woreta Town [22] that reported 40.9% and 59% prevalence of current and life time prevalence of alcohol use, while a similar study conducted in Sri Lanka reported a lower prevalence of alcohol uses (3.4%) [35]. The current and lifetime prevalence of khat chewing was 16.6% and 17.8%. This result is lower than the result of a study done among Haramaya University students [13] 23.6% and 41%, respectively, a study done in preparatory students in Woldia Town [21] 23.5%, a study in Rift Valley University 35.6% [37], and a study conducted in Saudi Arabia that reported 48.8% of lifetime khat chewing and 33.9% current khat chewing [39]. This variation may be because the accessibility of substances and difference in culture from region to region.

The magnitude of the prevalence lifetime and current cigarettes smoking were almost similar 4.8% and 4.5%, respectively. This finding is lower than a study in Woldia Town 23.5% [21] and a study in Addis Ababa University students 9% [15], and a study in Rift Valley University 18.4% [37], but higher than a study in Sri Lanka 2.3% [35]. This small prevalence in this study may be attributed to underreporting due to self-reporting or it might be because of the differences in study setting and population. The lifetime prevalence of illicit drug uses for this particular study is 16.4% the prevalence of current illicit drug uses is 8.4%. The finding of this study is almost with a study conducted in Haramaya University [13] that reported 17.4% of lifetime use of illicit drugs and 7.4% of current use of illicit substance use.

In the multivariable logistic regression analysis, the study found significant associations between substance and sex, living through high levels of family conflict, poor family management, age, friends' use of substance, poor academic performance, low school commitment, and illiterate education level of mother. Similarly, exposure to sexually explicit materials was strongly associated with sex, age, friends watching SEM, father education level, mother occupation, family's economic status, and chewing khat. Substance use was highly probable among male students compared to females. Male students were more likely to use substances than females. This finding agrees with earlier researches [13, 15, 20, 38] while some researchers found there is no significant association with substance and sex [18, 21]. In this study, students whose friends use substance were more likely to use substances as compared to that of whose friends did not use. This finding agrees with previous studies conducted in Addis Ababa University [15], Woreta Town [22], Woldia Town [21], Wolaita Sodo University [40], and Addigrat University [23].

This study shows that substance use is associated with poor family management and living through high level of conflict. Students who are living through high level of conflicts are more likely to use substances. Findings from previous research [41] suggest using substances was higher among adolescents living through high level of conflict. Poor family management was also directly associated with adolescents using substance, where adolescents that belong to poor family managements have a higher odd of using substances. This finding was consistent with the result of [42, 43].

Poor academic performance was another variable identified as a significant predictor of substance uses among adolescents in the multivariate analysis. The findings show that adolescents with poor academic performance have a significantly higher odds of substance compared to those with good academic performance. This finding was consistent with the result of [39, 42] that lower achievers had a high likelihood of using substances. Additionally, low school commitment and mother's education level was found to be significant covariates of substance use. Adolescents from schools with low commitment to control drugs and follow students more likely to use substance [44] while [45] found that there is no association with school commitment and substance use. Adolescents belong to mothers' with no education at all had a higher odds of using substance compared to those belongs to mothers of higher education. This finding is in agreement with prior studies in Korea [46] which shows a significant association with maternal education and drug uses. Age of adolescents was another variable significantly associated with substance use. The odds of substance uses were higher among adolescent 17–19 years old compared to that of 14–16 years. This finding is congruent with a study conducted in Sri Lanka [35, 47, 48].

This study also found that friends' use of sexually explicit material has increased rates of exposure to sexually explicit materials among adolescents, which was congruent with previous studies [49]. This event may be due to high peer pressure and initiation to be sociable with friends. Sex was another variable found to be significantly associated with adolescents' exposure to sexually explicit materials. The rate of exposure to sexually explicit material was higher among male adolescents than females. A previous studies had reported that being male was associated with increased risk of exposure to SEM than females [49–53]. Exposure to sexually explicit materials was also inversely associated with family economic status. This may due the fact that children belonging to rich family had access to different Internet and videos discs. This finding was consistent with earlier studies [52]. Mothers' occupation was also found to significantly associate with adolescents exposure to SEM. Higher odds of exposure to sexually explicit materials existed among mothers who were not working and self-employed. This finding is consistent with a study conducted among preparatory students in Addis Ababa [52].

Khat chewing was also an important factor that significantly related to adolescents exposure to sexually explicit materials. It is vital that use of khat and other illicit drugs initiate adolescents to read/watch pornographic materials. The findings of this study showed that adolescents who chew khat were probably highly exposed to SEM than those who had not chewing khat. Existing literature also indicates that khat chewing and using drug had a higher access to get exposure to SEM [49, 50, 53]. The findings of the study also revealed that adolescents 17–19 years old were more likely exposed to SEM. A study conducted by [54, 55] indicated that adolescents age has a significant association with sexually explicit content on the Internet. Exposure to sexually explicit materials was also significantly higher among adolescents born to a father with a secondary education compared to that of a diploma or higher. This finding is congruent with a study by [56].

The study did not find a significant association between grade levels, access of substance near school, family history of substance use, family economic status and substance use, which was inconsistent with prior studies on determinants of substance use [13, 18, 22]. Peer pressure, poor family management, academic performance, mother's education level, use of alcohol, and use of illicit substances were not significantly associated exposure to SSEM. However, this finding differs from earlier studies [49–51].

## 6. Conclusion

The study has examined the prevalence and factors associated with high-school adolescents' substance use and exposure to sexually explicit materials in North Shewa zone Oromia region using logistic regression analysis. The finding of this study supports that the prevalence of substance use and exposure to sexually explicit materials among high school adolescents is still high and is an important public health issue in the study area. In the results of multivariable logistic regression analysis, it was found that being male sex, living through high levels of family conflict, poor family management, friends' use of substance, poor academic performance, adolescents age 17–19 years, and low school commitment were factor significantly increase the risk of substance uses. On the other hand, being male sex, adolescents age 17–19 years, having friends watching sexually explicit materials, father's education level, family's economic status, and chewing khat were factors strongly positively correlated with high school adolescents exposure to sexually explicit materials.

To achieve the sustainable development goal indicator, 3.5 targeted to strengthen the prevention and treatment of substance abuse, interventions that focus on family management, peer pressure, and school commitment are required to decrease the prevalence of substance uses and exposure to sexually explicit materials among high-school adolescents.

## 7. Strength and Limitations of the Study

This study used a questionnaire adapted from the standard the 2008 “Community That Care Youth Survey” for adolescent substance use and problem behaviors. The major limitation was that misreporting or underreporting of illicit drugs uses. Also the study included only students studying in school. The other limitation was that interaction effects were not used included in the analysis due to large number of variables to compute.

## Figures and Tables

**Table 1:** Socioeconomic, demographic and behavioral characteristics of adolescents.

Characteristics	Categories	*N*	%
Sex	Male	230	61
	Female	147	39

Grade	9^th^	164	43.5
	10^th^	213	56.5

Living with	Oneself	61	16.9
	Parent	256	71.1
	Relatives	31	8.6
	Guardians/others	12	3.3

Source of income	Oneself	52	14.4
	Parent	264	79.2
	Relatives	27	7.5
	Guardians/others	19	5.2

Academic rank in the recent semester	1–10	240	65.6
	11–20	89	24.3
	21–30	15	4.1
	>30	22	6

Religion	Coptic orthodox	322	87.4
	Muslim	16	4.2
	Protestant	20	5.3
	Others	12	3.2

Frequency of attending religious program	Daily	145	39.2
	Once a week	118	31.9
	More than twice a week	56	15.1
	Once in two week	19	5.1
	Once a month	14	3.8
	Once in 6 months to one year	18	4.9

Mother education level	Illiterate	148	40.3
	Read and write	80	21.8
	Primary school	61	16.6
	Secondary school	37	10.1
	Higher education	41	11.2

Father education	Illiterate	105	28.5
	Read and write	93	25.2
	Primary school	60	16.3
	Secondary school	39	10.6
	Higher education	72	19.5

Mother occupation	Unemployed	79	22.1
	Employed	50	14
	Self-employed	189	52.9
	Daily laborer	15	4.2
	Others	24	6.7

Father occupation	Unemployed	45	12.9
	Employed	78	22.3
	Self-employed	177	50.7
	Daily laborer	21	6
	Others	28	8

Family economic status	Poor	76	20.5
	Moderate	238	64.3
	Rich	56	15.1

**Table 2:** Lifetime and current use of different substances by sex among high school adolescents in North Shewa zone.

Substance	Sex		Total (%)
*Any substance*	Male (%)	Female (%)	
Yes	110 (61.1)	70 (38.89)	180 (47.75)
No	120 (60.91)	77 (39.1)	197 (52.25)

*Ever use of khat*			
Yes	41 (64.1)	23 (35.9)	64 (17.8)
No	175 (59.3)	120 (40.7)	295 (82.2)

*Current use of khat*			
Yes	39 (63.93)	22 (36.07)	61 (16.6)
No	185 (60.26)	122 (39.74)	307 (83.4)

*Ever use of alcohol*			
Yes	90 (57.69)	66 (42.31)	156 (42.16)
No	135 (63.08)	79 (36.92)	214 (57.84)

*Current use of alcohol*			
Yes	69 (72.6)	26 (27.4)	95 (26.1)
No	153 (56.9)	116 (43.1)	269 (73.9)

*Ever use of cigarette*			
Yes	12 (66.67)	6 (33.33)	18 (4.8)
No	208 (66.88)	130 (33.12)	311 (95.2)

*Current use of cigarette*			
Yes	10 (62.5)	6 (37.5)	16 (4.5)
No	210 (61.2)	133 (38.8)	343 (95.5)

*Ever use of illicit drugs*			
Yes	40 (66.67)	20 (33.33)	60 (15.9)
No	190 (59.94)	127 (40.06))	317 (84.1)

*Current use of illicit drugs*			
Yes	22 (59.5)	15 (40.5)	37 (10.3)
No	194 (60.2)	128 (39.8)	322 (89.7)

**Table 3:** Major motivations and reasons to use substances.

	N	%
*Basic motivations to use drugs*		
Peer pressure	30	9.1
Availability of drugs	29	9
Academic failure	41	13.4
Dissatisfaction with the school environment	36	11.7
Having biological relatives with drug abuse	39	12.4
Unhappiness at home	42	13.4
To cope with various life challenges	47	15.1
To experience pleasure	58	18.8
To socialize	69	22.5

*Major reason for khat chewing*		
To pass the time	20	30.8
To stay awake	8	12.3
To socialize	9	13.8
Being addicted	11	16.9
Others	17	26.2

*Major reason for using alcohol*		
To pass the time	34	27.2
Depression	8	6.4
To forget problems	13	10.4
To socialize	17	13.6
Being addicted	10	8
Others	43	34.4

**Table 4:** Overall model goodness of fit checking using likelihood test and Hosmer–Lemeshow test.

Model 1	−2log likelihood	Likelihood ratio test	df	*p* value
Null model	187.474	58.27	29	0.001
Full model	129.204			

Hosmer–Lemeshow test = 10.196 (*p* value = 0.253)				

Model 2	−2log likelihood	Likelihood ratio test	df	*p* value
Null model	184.241	49.395	28	<0.001
Full model	134.846			
Hosmer–Lemeshow test = 11.333 (*p* value = 0.184)				

**Table 5:** Major consequences of substance.

Consequences of substance	N	%
*Health aspects*		
Physically ill-health	29	35.8
Sleep disorder	9	11.1
Mental illness	13	16
Others	30	37.1

*Social aspects*		
Loosing friends	19	22.6
Problems with parents/guardians	33	39.3
Being arrested	6	7.1
Others	26	31

*Behavioral aspects*		
Lateness from school	15	12.5
Violence (disciplinary problems)	18	15
Absenteeism from school	20	16.7
Loss of interest in daily activities	8	6.7
Others	59	49.7

*Psychological aspects*		
Psychological distress	53	54.1
Suicide attainment	11	11.2
Others	44	34.7

**Table 6:** Factors associated with life time substance use among high school adolescents in North Shewa zone.

Variables	Substance use		AOR	95% CI for AOR	*p* value
	*N*	%			
Sex					
Male	110	61.1	2.334	1.549, 9.926	0.002^*∗*^
Female	70	38.9	1		

*Living through high levels of family conflict*					
Yes	29	17.4	6.25	1.745, 10.00	0.023^*∗*^
No	138	82.6	1		

*Poor family management*					
Yes	42	25.1	27.084	1.624, 45.56	0.022^*∗*^
No	125	74.9	1		

*Pressure from my friends to use substance*					
Yes	41	23.8	12.882	1.882, 88.153	0.009^*∗*^
No	131	76.2	1		

*Friends use of substances*					
Yes	25	14.3	11.928	1.761, 18.692	0.007^*∗*^
No	150	85.7	1		
Age					
14–16	35	24.3	0.261	0.100, 0.683	0.006^*∗*^
17–19	109	75.7	1		

*Access of substances near your school*					
Yes	41	26.3	1.062	0.128, 8.787	0.956
No	115	73.7	1		

*Poor academic performance*					
Yes	35	21.6	14.48	1.290, 162.58	0.030^*∗*^
No	127	78.4	1		

*Low school commitment*					
Yes	39	24.5	11.951	1.418, 100.73	0.033^*∗*^
No	120	75.5	1		

*Family history of substance use*					
Yes	32	19.6	5.915	0.748, 46.773	0.092
No	131	80.4	1		

*Family economic status*					
Poor	39	22	0.405	0.033, 5.052	0.483
Moderate	110	62.1	3.699	0.500, 27.358	0.200
Rich	28	15.8	1		

*Mother's education*					
Illiterate/read and write	117	65.7	2.051	0.068, 61.564	0.051
Primary	33	18.5	2.165	0.074, 62.981	0.653
Secondary	13	7.3	1.584	0.044, 56.408	0.053
Diploma/higher	15	8.4	1		

*Father's education*					
Illiterate/read and write	107	61.1	2.936	0.553, 15.588	0.206
Primary	23	13.1	2.868	0.256, 28.212	0.410
Secondary	20	11.4	0.053	0.002, 1.410	0.053
Diploma/higher	25	14.3	1		

**Table 7:** Factors associated with high school adolescents' exposure to sexually explicit materials in North Shewa zone.

	Watch SEM		AOR	95% CI for AOR	*p* values
	*N*	%			
Sex					
Male	96	71.1	7.52	2.611, 21.739	<0.0001^*∗*^
Female	36	28.9	1		

*Grade*					
9^th^	57	44.2	1.214	0.521, 2.831	0.653
10^th^	72	55.8	1		

*Positive parental role modeling*					
Yes	44	34.9	0.519	0.177, 1.523	0.232
No	82	65.1	1		

*Peer pressure*					
Yes	34	25.6	1.986	0.502, 7.865	0.328
No	99	74.4	1		

*Age*					
14–16	34	32.7	0.201	0.071, 0.565	0.002^*∗*^
17–19	70	67.3	1		

*Friends watch SEM*					
Yes			5.376	1.010, 28.571	0.049^*∗*^
No			1		

*Poor academic performance*					
Yes	33	30	0.491	0.154, 1.561	0.228
No	77	70	1		

*School commitment*					
Yes	29	26.6	1.413	0.364, 5.479	0.617
No	80	73.4	1		

*Mother education status*					0.163
Illiterate	58	43	0.585	0.052, 6.619	0.665
Read and write	36	26.7	0.588	0.053, 6.496	0.665
Primary school	17	12.6	1.219	0.098, 15.151	0.878
Secondary school	14	10.4	0.136	0.012, 1.543	0.107
Diploma/higher	10	7.4	1		

*Father education status*					
Illiterate	41	31.1	0.651	0.130, 3.274	0.603
Read and write	42	31.8	0.378	0.085, 1.682	0.202
Primary school	19	14.4	1.753	0.293, 10.479	0.538
Secondary school	11	8.3	7.996	1.010, 63.306	0.049^*∗*^
Diploma/higher	19	14.4	1		

*Mother's occupation*					0.022^*∗*^
Not working	28	22	25.151	3.290, 192.293	0.002^*∗*^
Employed	15	11.8	4.804	0.598, 38.626	0.140
Self-employed	61	48	6.577	1.013, 42.721	0.048
Daily laborer	8	6.3	7.436	0.539, 102.66	0.134
Others	15	11.8	1		

*Family economic status*					0.036^*∗*^
Poor	21	15.9	8.433	1.647, 43.18	0.011^*∗*^
Moderate	83	62.9	3.948	1.088, 14.327	0.037^*∗*^
Rich	28	21.2	1		

*Chew khat*					
Yes	45	33.3	12.5	2.924, 52.632	0.001^*∗*^
No	90	66.7	1		

*Use alcohol*					
Yes	71	51.4	0.344	0.036, 3.291	0.354
No	67	48.6	1		

*Use other illicit substance*					
Yes	44	31.9	1.182	0.130, 10.766	0.882
No	94	68.1	1		

## Data Availability

The data used to support the findings of this study are available from the corresponding author upon request.
